# An Attention-Guided Spatiotemporal Graph Convolutional Network for Sleep Stage Classification

**DOI:** 10.3390/life12050622

**Published:** 2022-04-21

**Authors:** Menglei Li, Hongbo Chen, Zixue Cheng

**Affiliations:** 1 Graduate School of Computer Science and Engineering, The University of Aizu, Tsuruga, Ikki-machi, Aizu-Wakamatsu City 965-8580, Fukushima, Japan; d8241102@u-aizu.ac.jp; 2 School of Computer Science and Engineering, The University of Aizu, Tsuruga, Ikki-machi, Aizu-Wakamatsu City 965-8580, Fukushima, Japan; z-cheng@u-aizu.ac.jp

**Keywords:** sleep stage classification, spatiotemporal graph convolutional network, attention

## Abstract

Sleep staging has been widely used as an approach in sleep diagnoses at sleep clinics. Graph neural network (GNN)-based methods have been extensively applied for automatic sleep stage classifications with significant results. However, the existing GNN-based methods rely on a static adjacency matrix to capture the features of the different electroencephalogram (EEG) channels, which cannot grasp the information of each electrode. Meanwhile, these methods ignore the importance of spatiotemporal relations in classifying sleep stages. In this work, we propose a combination of a dynamic and static spatiotemporal graph convolutional network (ST-GCN) with inter-temporal attention blocks to overcome two shortcomings. The proposed method consists of a GCN with a CNN that takes into account the intra-frame dependency of each electrode in the brain region to extract spatial and temporal features separately. In addition, the attention block was used to capture the long-range dependencies between the different electrodes in the brain region, which helps the model to classify the dynamics of each sleep stage more accurately. In our experiments, we used the sleep-EDF and the subgroup III of the ISRUC-SLEEP dataset to compare with the most current methods. The results show that our method performs better in accuracy from 4.6% to 5.3%, in Kappa from 0.06 to 0.07, and in macro-F score from 4.9% to 5.7%. The proposed method has the potential to be an effective tool for improving sleep disorders.

## 1. Introduction

**Figure 1:**
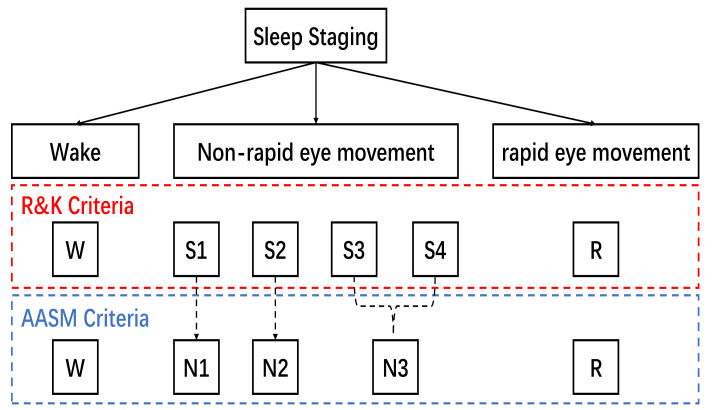
Terminology used by R&K and AASM for sleep stage classification. In R&K criteria, the sleep stage is classified into *W* (wake), $S_{1}$, $S_{2}$, $S_{3}$, $S_{4}$, and *R* (rapid eye movement). In AASM criteria, $S_{3}$ and $S_{4}$ are merged into a single stage $N_{3}$.

Sleep is an indispensable physiological phenomenon for human beings, which acts as preventive medicine for physical and mental diseases and mood improvement [1]. However, due to social competition, work pressure, and the accelerated aging of the population, sleep disorders have become health risks that cannot be ignored; these disorders are mainly manifested as insomnia, circadian rhythm disorders, and obstructive sleep apnea (OSA) syndrome [2,3]. The incidence and characteristics of various sleep disorders vary at different sleep stages. In order to make diagnoses, sleep specialists have introduced polysomnogram (PSG) [4] to monitor and record data from the body. PSG is a biological signal obtained through various sensors on different parts of the body, including an electroencephalogram (EEG), electrooculogram (EOG), electromyogram (EMG), and electrocardiogram (ECG). EEG is a cost-effective and, typically, a non-invasive test for monitoring and recording electrical activity during sleep. Moreover, EMGs and EOGs have been used as two important switches for detecting rapid eye movement (REM) sleep [5]. Therefore, human experts need to combine other biological signals (such as EEG, EOG, and EMG) to achieve manual sleep stage classification. Rechtschaffen and Kales (R&K) [6] delineated six sleep stages during sleep using early PSG. They categorize non-rapid eye movements (NREMs) into four sleep stages ($S_{1}$, $S_{2}$, $S_{3}$, and $S_{4}$). For standardization, the American Academy of Sleep Medicine (AASM) [7] has defined the sleep staging criteria to achieve sleep scoring. According to the AASM manual, sleep experts use consecutive 30-s epochs of PSG data to classify five stages. These are wake, rapid eye movement (also referred to as stage *R*), and three NREMs, $N_{1}$, $N_{2}$, and $N_{3}$. Based on the R&K criteria or the AASM criteria, sleep stages are shown in Figure 1. Manual sleep stage classification is a laborious task [8]. Therefore, automatic sleep stage classification with rapid and high accuracy based on EEG signals is of great research interest.

Looking back on the past decades, the various methods in the relevant studies on sleep stage classification have been proposed. According to studies [1], sleep stage research has far-reaching implications for biomedical practice. In the early days, researchers used the hand-engineered feature-based methods to extract features in the time and frequency domains for sleep stage analysis. For example, Tsinalis et al. [9] made the precision of sleep stage classification up to 78.9% via the extracted features in the time-frequency domains. Lee et al. [10] developed an automatic sleep staging system with a mean percentage agreement of 75.52% for diagnosing OSA, using single-channel frontal EEG to classify wake, light sleep, deep sleep, and REM sleep. In order to achieve sleep stage classification, some machine learning-based methods [11,12] have been introduced in sleep stage classifications, e.g., support vector machine (SVM) [13] and random forest [14]. However, these methods have some limitations, such as the need to observe each PSG epoch for extracting features with a prior knowledge. For the time being, more studies are focusing on deep learning-based methods. Owing to the availability of high-quality datasets of EEG signals, deep learning-based methods are widely used to extract features from EEG signals for sleep stage classification. In our opinion, the latest deep learning-based methods for sleep stage classification can be split into two categories: non-GCN-based methods and GCN-based methods.

1. Non-GCN-based Methods
  More studies are solving the task of sleep stage classification based on recurrent neural networks (RNNs) and convolutional neural networks (CNNs). RNNs are commonly used to model the temporal dynamics of EEG signals [15]. In SeqSleepNet [16], a hierarchical RNN is used to model sleep staging and achieve accuracy up to 87.1%. In RNN, there are two kinds of the most representative structures, long short-term memory (LSTM) [17] and gated recurrent unit (GRU) [18]. For example, IITnet [19] is proposed to automatically score sleep stages via BiLSTM. However, the problem of gradient disappearance or explosion occurs during RNN training, which makes it difficult to train a deep RNN model. Compared to RNNs, CNNs have high performance in parallel computing. To extract local and global features, Tsinalis et al. [20] proposed an automatic classification approach for sleep stage scoring based on single-channel EEG. Phan et al. [21] used a simple yet efficient CNN to extract sleep features from EEG signals. In addition, SleepEEGNet [22] employs deep CNNs as the backbone network for sleep stage classification, achieving an accuracy of 84.26 %. Chanbon et al. [23] introduce an end-to-end deep learning approach for sleep stage classification using multivariate and multimodal EEG signals. Furthermore, there are some works that combine CNN with RNN to simultaneously extract spatial and temporal features for sleep stage classification, e.g., DeepSleepNet [24] and TinySleepNet [25]. However, EEGs are non-Euclidean data, which naturally results in CNNs and RNNs being limited in feature extractions. Furthermore, their development potential is further hindered by the enormous parameter overhead.
2. GCN-based methods.
  The graph convolutional network (GCN) [26] is an advanced neural network structure for processing graph structured data. Since EEG channels are structured data with temporal relations, each channel can be considered as a node in the graph. For this reason, GCN-based methods have been proven to be more powerful in processing EEGs. Joint analysis of EEG and eye-tracking recordings is raised by Zhang et al. [27], whose strategy is to introduce GCN to fuse features. However, EEG channel signals include the temporal dynamic information of brain activity and the functional dependence between brain regions. To remedy the deficiency of the traditional spatiotemporal prediction model, the spatiotemporal graph convolutional network (ST-GCN) [28] is proposed to model spatiotemporal relations and to learn the dynamic EEG for the task of sleep staging. For example, the GraphSleepNet [29] is proposed to utilize brain spatial features and transition information among sleep stages to achieve more specific performance. However, the dependence on non-adjacent electrodes placed in different brain regions is often overlooked. Since then, Jia et al. [30] propose an multi-view spatial-temporal graph convolutional network (MSTGCN) to extract the most relevant spatial and temporal information with superior performance. They introduce spatiotemporal attention to extract temporal and spatial information, respectively. However, this method makes it ineffective to capture the spatiotemporal dependencies on separated attention.

After summarizing the previous works, there are three shortcomings that need to be solved: (1) topological connections of electrodes in context are not well captured; (2) these methods force GCNs to aggregate features in different channels with the same topology, which limits the upper bound of model performance; and (3) attention weights are not sufficient to summarize long-range spatiotemporal characteristics. In order to address the aforementioned challenges, we propose a combination of dynamic and static ST-GCN with inter-temporal attention blocks for automatic sleep stage classification.

Overall, the main contributions of our proposed approach can be summarized as follows:

- In previous work, sleep stage classification is achieved by complex modeling. In contrast, our proposed method is to leverage spatial graph convolutions along with interleaving temporal convolutions to achieve spatiotemporal modeling, which can be simpler yet efficient.
- The inter-temporal attention blocks are introduced to achieve an automatic sleep stage classification, which can withdraw the most informative information across space and time, further proving that capture spatiotemporal relation plays an important role in sleep stage classification.
- The proposed model significantly outperforms state-of-the-art methods on the sleep-EDF and the subgroup III of the ISRUC-SLEEP dataset. Our proposed method achieves better performance with 91.0% and 87.4% accuracy, both outperforming the state-of-the-art methods (86.4% and 82.1%).

The rest of this paper is organized as follows: In Section 2, we present a series of preparatory works for our study. In Section 3, we briefly describe the proposed network framework, including the dynamic and static ST-GCN and the inter-temporal attention block. The dataset used, the experiments, and the experimental results are presented in Section 4. Moreover, finally, we conclude this work and provide an outlook on future work in Section 5.

## 2. Preliminaries

A sleep stage network is described as an undirected graph G=(V,E), where $V=\left\{V_{1},V_{2},\cdots ,V_{n}\right\}$ is the collection of *N* nodes representing electrodes in the brain, and the edge set *E* represents the connection between nodes captured by an adjacency matrix A∈N×N. *A* is a matrix composed of 0 and 1, where 1 represents that the corresponding electrodes are connected, and 0 otherwise. Graph *G* is made up of a 30-s EEG signal sequence $S_{t}$. The sleep feature matrix is the input of *G*. We define the raw signal sequence as $S=\left\{S_{1},S_{2},\cdots ,S_{m}\right\}\in R^{m\times Q\times T}$, where *m* denotes the number of samples, *Q* means the number of electrodes, and *T* is the time series length of each sample $S_{i}\in S\left(i\in \left\{1,2,\cdots ,m\right\}\right)$. Inspired by Hyvräinen’s work [31], we can extract the features of differential entropy (DE) on different frequency bands and define them on each sample feature matrix. Therefore, we can obtain a feature matrix at each sample *i*, denoting the $F_{de}$ features of the nodes *N*.

(1) $$X_{i}={\left(x_{1}^{i},x_{2}^{i},\cdots ,x_{N}^{i}\right)}^{T}\in R^{N\times F_{de}}$$

Therein, $x_{n}^{i}\in R^{F_{de}}\left(n\in \left\{1,2,\cdots ,N\right\}\right)$ denotes the $F_{de}$ features of electrode node *n* at sample *i*.

The objective of our study is to establish a mapping relationship between sleep signals and sleep stages using a spatiotemporal neural graph network. The issue of sleep stage is described as follows:

(2) $$C=\left(X_{1},X_{1+d},\cdots ,X_{1+kd}\right)\in R^{N\times F_{de}\times T_{n}}$$

The given Equation (2) can identify the current sleep stage *S*. Therein, C denotes the temporal context of $X_{1+kd}$, *S* denotes the sleep stage class label defined by $X_{1+kd}$, $T_{n}$ indicates the length of sleep stage networks, *d* denotes the temporal context coefficient, and *k* is the number of intercepted time segments in a continuous EEG signal.

## 3. Methods

In this section, we introduce the components of our proposed network of sleep stage classification in detail.

### 3.1. Network Architecture

**Figure 2:**
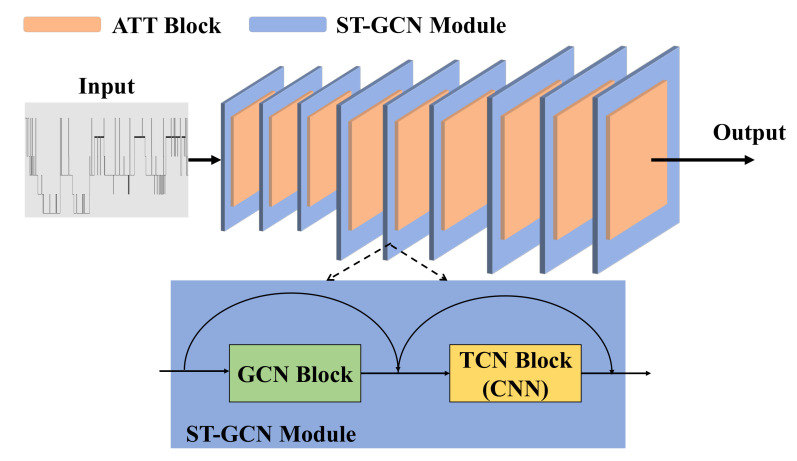
The proposed network architecture for sleep stage classification. The network consists of nine ST-GCN modules, each followed by an attention (ATT) block. Each ST-GCN module contains a GCN block followed by a TCN block. The numbers of output channel for ST-GCN modules are 66, 66, 66, 132, 132, 132, 264, 264, 264.

Figure 2 illustrates our network architecture. Inspired by spatiotemporal graph convolutional networks (ST-GCN) [28], we construct the network of sleep stage classification by nine serial connected ST-GCN modules that can extract more detailed feature information. The ST-GCN module contains a sequential execution of a GCN block and a temporal convolutional network (TCN) block. The TCN block is a one-dimensional CNN used for sequence modeling tasks. The GCN block and the TCN block in GCN aggregate features along the spatial dimension and the temporal dimension, respectively. Each ST-GCN module is followed by an attention block (ATT). The function of the ATT block allows the network architecture to pay more attention to important features of the sleep stage, thus better capturing spatiotemporal relations. As far as we know, this is the first attempt to introduce attention enhancement and spatiotemporal separated feature extraction together for sleep stage classification using EEGs. Each module is presented separately in the following subsections.

### 3.2. Graph Convolutional Network Module

In our work, we construct a spatiotemporal graph with the electrodes in the brain as graph nodes and natural connections in different brain region electrodes and time as graph edges. In sleep stage classification tasks, it is important that we model the spatial dependencies in the sleep stage network. GCN is able to effectively extract key point information from the spatiotemporal graph. To capture the dependency created by the topological structures of the electrodes in the context, the layer-wise update rule of GCNs may be implemented to features at time *T* on sleep inputs defined by features *X* and the graph structure $\tilde{A}$, as follows:

(3) $$X_{T}^{l+1}=\lambda \left({\tilde{D}}^{-\frac{1}{2}}\tilde{A}{\tilde{D}}^{-\frac{1}{2}}X_{T}^{\left(l\right)}\mu^{\left(l\right)}\right)$$

Therein, $\tilde{D}$ is the diagonal degree matrix of $\tilde{A}$, and the sleep graph with self-loops $\tilde{A}=A+I$ consists of an adjacency matrix *A* and an identity matrix *I*. This allows $\tilde{A}$ to preserve the identity features. The $\lambda \left(\cdot \right)$ is an activation function and the μ denotes the weight matrix. Moreover, ${\tilde{D}}^{-\frac{1}{2}}\tilde{A}{\tilde{D}}^{-\frac{1}{2}}$ can be conceived as an approximate spatial mean feature aggregation from the immediate neighborhood followed by an activated linear layer.

In static methods, $\tilde{A}$ is defined manually or set as a trainable parameter. The topology is predefined according to the structure, and is fixed in both the training and testing phases. Notably, these methods have some limitations, such as the need for a prior knowledge and the inability to construct dynamic graph topologies. To overcome these limitations, the model is usually required to be generated adaptively depending on the input sample. Therefore, a dynamic ST-GCN [29] is proposed that defines a non-negative function to represent the connection relationship between electrode nodes $N_{i}$ and $N_{j}$ based on the input feature matrix. From this effect, the dynamic adjacency matrix is more powerful since it can be dynamically adapted during the training process and has a stronger generalization ability compared to static methods due to the dynamic topologies. Although the use of dynamic topologies leads to good performance, the original structural information is often discarded. Therefore, we propose a combination of dynamic and static GCN that incorporates contextual features of all brain regions to learn correlations between arbitrary pairs of points.

In the static branch, we use the physical graph $G_{p}$ from the physical connections of the electrode structure and the parameterized mask $G_{m}$ is used to pay attention to the physical graph $G_{p}$. The static topology information of the electrode structure is extracted in the static branch, which has been shown to be useful for the final prediction. The output of the static branch can be shown as follows:

(4) $$Output_{static}=\lambda \left(G_{p}+G_{m}\right)X_{T}^{\left(l\right)}\mu^{\left(l\right)}$$

In the dynamic branch, the predicted dynamic graph $G_{d}$ is used as input. The output of the dynamic branch extracts the global context-enriched topology of the electrode structure. We represent the output of the dynamic branch as:

(5) $$Output_{dynamic}=G_{d}X_{T}^{\left(l\right)}\mu^{{\left(l\right)}^{{}^{\prime }}}$$

therein, the learnable kernel $\mu^{{\left(l\right)}^{{}^{\prime }}}$ is not shared between the static and dynamic branches. Moreover, we fuse static and context-enriched topology features extracted by the static and dynamic branches using a weighted summation method. It can be expressed as:

(6) $$Output=Output_{dynamic}+\phi Output_{static}$$

where ϕ goes from 0 to 1, which is a balance between the output of the static and dynamic branches.

### 3.3. Multi-Scale CNN Module

Temporal modeling is essential to sleep stage classification as well. Studies [32,33,34] show that RNNs achieve great performance in temporal modeling tasks. However, the main shortcomings of RNNs are time, cost, and its inability to retain long-term memory. Namely, RNNs cannot perform massively parallel processings like CNNs. TCN [35], as a temporal variant of CNN, has promising performance in time series forecasting. Since sleep stage classification is time-dependent, TCN is used to capture dependencies between sleep stages for achieving sleep stage classification. Multi-scale convolutional neural networks can adaptively fuse multi-scale temporal features extracted by different scale convolution kernels. Thus, they can better model temporal topological features.

**Figure 3:**
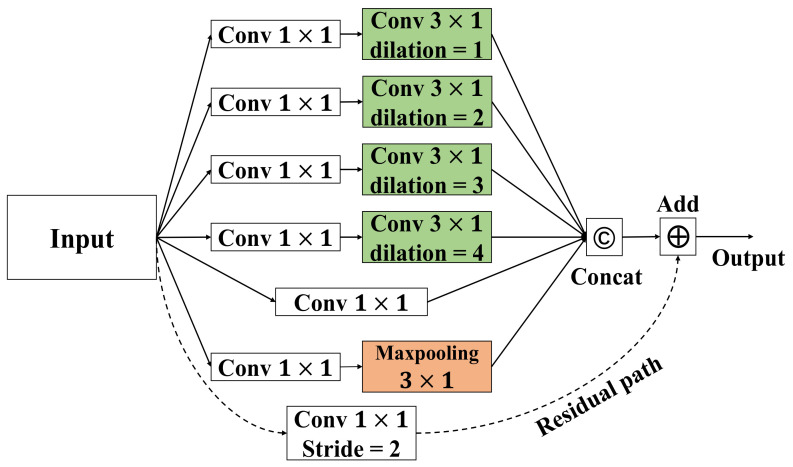
Multi-scale convolutional neural network architecture.

In order to achieve temporal modeling, many previous studies [36,37,38] have used temporal convolutions with a fixed kernel size $k_{t}\times 1$ throughout the architecture. As a natural extension to the multi-scale spatial aggregation, we used multi-scale learning to improve vanilla temporal convolutional layers, as shown in Figure 3. To reduce the computational costs incurred by the extra branches, we introduce the idea of a bottleneck design [39], set the kernel size to 3 × 1, and employ different dilation factors [40] instead of larger kernels for larger receptive fields to construct a multi-scale time-series layer. Specifically, seven temporal convolution branches are arranged in parallel. Each branch uses a bottleneck structure (i.e., 1×1 convolution) to reduce the number of feature channels and the calculation amount, thus accelerating the training speed and model inference. Moreover, as the input passes forward, the functions of distinct branches diverge, which can be divided into the following four types.

- Multi-scale temporal feature extraction: in the four temporal convolution branches, each branch consists of 3×1 temporal convolutions. Each 3×1 temporal convolution uses different dilations to obtain multi-scale temporal receptive fields.
- Feature processing within the current frame: this second type only has a temporal convolution with the kernel size 1 × 1 to concentrate features within a single frame.
- Emphasizing the most salient information within the consecutive frames: the third type is to be followed by a 3 × 1 max-pooling layer to draw the most important features.
- Gradient preservation: to preserve gradients during back-propagation, we add a residual path in the final type.

Finally, we use residual connections [41] to facilitate training.

### 3.4. Inter-Temporal Attention

**Figure 4:**
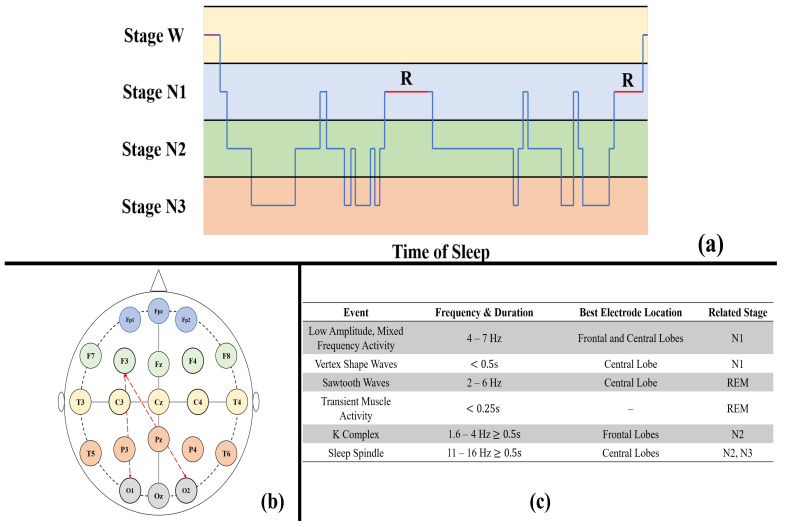
(**a**) An example of a profile of the sleep stages; (**b**) EEG electrode placement in the 10–20 system, and the *F*, *T*, *C*, *P*, and *O* denote frontal, temporal, central, parietal, and occipital lobe placements, respectively; (**c**) EEG waves and events during sleep [11].

Most existing approaches [28,38,42] use graph convolution to extract spatial relations at each time step and 1D convolutional layers to model temporal dynamics. However, these methods make it difficult to obtain the direct information flow across spacetime, and complex regional joint spatiotemporal dependencies are not captured. In other words, the factorized modeling cannot capture the long-range features with precise temporal information. In recent years, attention mechanisms have found wide application in various classification tasks, which have made remarkable achievements [43,44]. The essence of attention mechanisms is to select the relatively critical information from the input. In our work, we consider the spatiotemporal relation of the EEG data and the stability of the learned representations for different sleep stage sequences. For example, in sleep stages *R* and $N_{1}$, the topological features of adjacent electrodes are similar, as shown in Figure 4a,c. To extract strongly distinctive features, there is a need for long-range dependencies in time and precise temporal information in space. In the spatial dimension, the shorter the path length, the more efficient the information transfer between the two electrodes. We pass the relevant features of the distant (informative) electrode to the target electrodes with much higher weights. An example is given in Figure 4b. The feature weights of electrode $F_{3}$ are passed to electrodes $O_{1}$ or $O_{2}$, which can pay attention to important features of distant electrodes in classifying similar sleep stages and better achieve sleep stage classification. Moreover, each electrode is expressed by a time series. In the temporal dimension, there are similarities among neighboring sleep stages, and we attend to important time steps of each electrode. Therefore, the inter-temporal attention is introduced to capture the spatial and temporal correlations in the sleep stage classification network.

The classification tasks introduce attention mechanisms to improve the classification effects, which are mainly implemented by a multi-layer perception (MLP), such as the SENet structure [45]. These modules are usually executed independently for each channel or spatial dimension, while other dimensions are globally averaged into a single unit. Since there is a strong link between spatial and temporal information based on GCN in sleep stage classification. It is clear that features separated from frames and electrodes are sub-optimal for weighting the importance of electrodes in different sleep stages, owing to the fact that the spatiotemporal relations are ignored.

**Figure 5:**
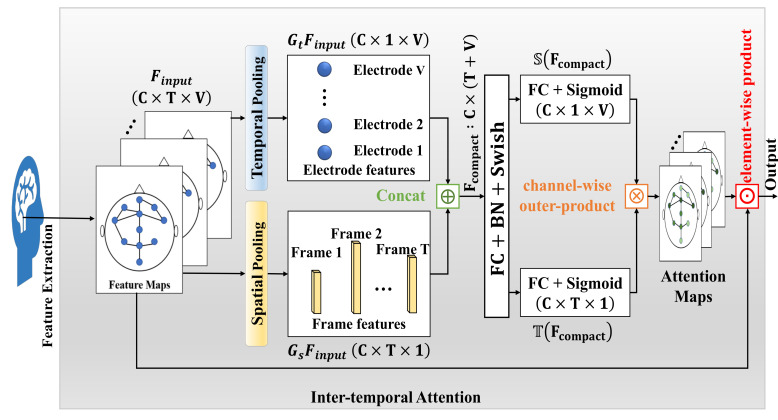
The overview of the inter-temporal attention block. *C*, *T*, and *V* denote the numbers of input channels, the length of the sequence, and the number of electrodes, respectively. BN denotes the batch normalization.

We separately consider that the frame and electrode are sub-optimal for weighting the importance of the electrode structure in the sleep stage classification. As an application of coordinate attention [46] for sleep stage classification, we propose an inter-temporal attention to enhance the model’s ability to extract informative features. It not only identifies the most informative points in certain frames from the whole input sequence, it can also help the network of sleep stage classification to capture richer features. Figure 5 is the overview diagram of the inter-temporal attention block. We present the details of an attention block in detail.

- We used a sequence of EEG signals as input, a sequence of EEG signals consists of T number of frames. Each frame consisted of sleep information with dimension C×V, where *V* is the number of electrodes and *C* is the number of channels. The input features ($F_{input}$) were passed through temporal pooling ($G_{t}$) and spatial pooling ($G_{s}$), respectively. After the operation of pooling, we aggregated the information in the frame- and electrode- dimension, yielding two sets of feature maps with temporal- and spatial-aware characteristics, the electrode features ($G_{t}F_{input}$), and the frame features ($G_{s}F_{input}$).
- We used the concatenation (⊕) operation to obtain the pooled feature vectors ($F_{compact}$), and used the fully connected layer (FC layer) to obtain the compact information. The activation function Swish (η) [47] is utilized in this FC layer.
- We used two relatively independent FC layers to recover the electrode features and the frame features into the same shape as the input separately. Then, applying the sigmoid activation function (τ) to the updated tensor. Hence, we can obtain two sets of attention scores, which are from the frame dimensions and the electrode dimensions, respectively. We used the attention scores to reweigh the raw feature maps in frame- and electrode- dimensions. Namely, the $T(F_{compact})$ and $S(F_{compact})$ denote the transfer matrix of the frame and electrode, respectively. In two independent FC layers, we multiplied the obtained attention scores for frame dimensions and electrode dimensions by the channel-wise outer-product (⊗).
- An element-wise product (⊙) was performed, resulting in output feature maps ($F_{output}$). The results of the multiplication could be considered as the attention scores for each electrode in the whole sleep cycle.

The inter-temporal attention module can be explained concisely and intuitively with the following two equations:

(7) $$F_{compact}=\eta \left(MLP\cdot \left(G_{t}F_{input}⊕G_{s}F_{input}\right)\right)$$

(8) $$F_{output}=F_{input}⊙\left(\tau \left(T\left(F_{compact}\right)⊗S\left(F_{compact}\right)\right)\right)$$

**Figure 6:**
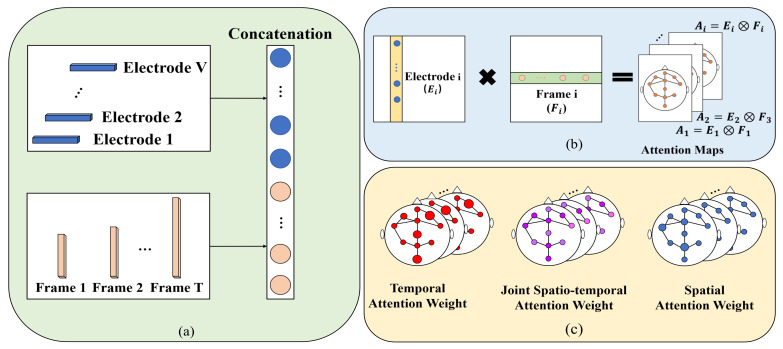
The details of our introduced inter-temporal attention block. (**a**) The pooled temporal and spatial feature vectors are concatenated; (**b**) outer product multiplication of frame- and electrode- matrices. Each electrode and the corresponding frame are multiplied with each other to product matrices *A*, attention maps; (**c**) example of obtaining the joint spatiotemporal attention weight. The inter-temporal attention blocks capture long-range features with precise temporal information.

To extract the most noteworthy information from the EEG signal sequence, we perform the max pooling operation under the frame- and electrode- dimensions, respectively. The max pooling plays a similar role as the attention mechanism, the maximum weight of the two dimensions can be selected by this operation. Then, the two groups of the obtained feature maps are concatenated, as shown in Figure 6a. We use the fully connected layer to squeeze the dimensions of the concatenated feature map. Thus, we obtain a continuous feature mapping for our subsequent extraction of the different dimensions of feature attention. After the split operation, two sets of attention scores for the frame dimension and the electrode dimension can be obtained, respectively. What we need is a relationship of attention across time and space, the attention scores of frames and electrodes are multiplied by a channel-wise outer-product, as shown in Figure 6b. Moreover, the result can be seen as the attention scores for each electrode in the whole EEG signal sequence. The attention score is a trainable inter-temporal signal. The joint spatiotemporal attention weight can be seen as the interaction of temporal attention weight and spatial attention weight, and we aggregate the temporal attention branch on the left and the spatial attention branch on the right, as shown in Figure 6c. Finally, we assign the generated spatiotemporal attention weights to the feature maps to obtain the attention responses across space and time. The most informative frames and electrodes can be more accurately located using the attention block, which helps the model to better complete sleep stage classification. As far as we know, this is the first time that inter-temporal attention blocks are introduced for automatic sleep stage classification.

## 4. Results

In this section, we evaluate the performance of the proposed method using the publicly available ISRUC-SLEEP dataset. The detailed description of the ISRUC-SLEEP dataset, sleep-EDF dataset, and the experimental setups can be given in the first two subsections. Then, we report the results of our proposed model compared to the other state-of-the-art models on the same dataset.

### 4.1. Dataset and Experimental Settings

To evaluate the performance of our method, we use the two publicly available datasets in this study: sleep-EDF dataset [48,49] and ISRUC-SLEEP dataset [50], which are the most widely used open-source datasets for state-of-the-art methods of sleep stage classification.

#### 4.1.1. Sleep-EDF Dataset

The sleep-EDF dataset records the EEG of 20 healthy Caucasian male and female subjects (ages 28.7 ± 2.9) without medication, and each EEG is sampled at 100 HZ from $F_{pz}$-$C_{z}$ and $P_{z}$-$O_{z}$ electrode locations. The EEG recording is manually classified into eight patterns (Wake, $S_{1}$, $S_{2}$, $S_{3}$, $S_{4}$, REM, movement, and unknown) according to the scoring rules of R&K [6]. In our experiment, we combine the $S_{3}$ and $S_{4}$ stages into one stage $N_{3}$ according to the AASM manual [7]. As the EEG is recorded over a long period of time, the stages movement and unknown are recorded at the beginning and end of each recording, when the subjects are awake. Therefore, movements (and unknown) are not used for sleep stage classification. Consequently, we obtain a dataset with five classes, including *W* (Wake), $N_{1}$ ($S_{1}$), $N_{2}$ ($S_{2}$), $N_{3}$ ($S_{3}$ + $S_{4}$) and *R* (REM). We use the 30-min EEG before and after the sleep period as experimental data.

#### 4.1.2. ISRUC-SLEEP Dataset

The ISRUC-SLEEP dataset from the Portuguese Foundation for Science and Technology (PFST) has three subgroups, with each subgroup recording the EEGs of 100 participants, 8 participants, and 10 participants, respectively. In order to compare healthy subjects with the patients suffering from sleep disorders, we used the subgroup III as the experimental dataset in our study; the EEG recordings of nine healthy male subjects and one healthy female subject aged between 30 and 58 years. Moreover, each EEG recording contained six EEG channels (i.e., $C_{3}$-$A_{2}$, $C_{4}$-$A_{1}$, $F_{3}$-$A_{2}$, $F_{4}$-$A_{1}$, $O_{1}$-$A_{2}$, and $O_{2}$-$A_{1}$) and is sampled at 200 Hz. The EEG recordings were visually scored by a human expert. According to the AASM manual [7], there were five classes in this dataset, including *W* (Wake), $N_{1}$, $N_{2}$, $N_{3}$, and *R* (REM). Table 1 shows the number of sleep stages in two different datasets.

### 4.2. Experimental Settings

We use the 20-fold cross-validation and 10-fold cross-validation to evaluate our method. In each iteration, we use the recordings of one subject as the test set, while the remaining one recording is considered as the training set. We implement our model with PyTorch 1.7.1, CUDA 11.4, and Anaconda 4.10.3. The hyperparameters of our experiment are listed in Table 2.

### 4.3. The Performance of Sleep Stage Classification

In our study, we use some metrics to evaluate the proposed model [51,52,53], e.g., the macro-precision, macro-recall, macro-F score, and Cohen’s Kappa coefficient. The macro-precision ($P_{macro}$), macro-recall ($R_{macro}$), macro-F score ($F_{macro}$), and Cohen’s Kappa coefficient (κ) are calculated as follows:

(9) $$P_{macro}=\frac{1}{n}\sum_{i=1}^{n}{\left(\frac{TP}{TP+FP}\right)}_{i}$$

(10) $$R_{macro}=\frac{1}{n}\sum_{i=1}^{n}{\left(\frac{TP}{TP+FN}\right)}_{i}$$

(11) $$F_{macro}=2\times \frac{P_{macro}\times R_{macro}}{P_{macro}+R_{macro}}$$

(12) $$\kappa =1-\frac{1-p_{o}}{1-p_{e}}$$

Therein, TP, FP, and FN stand, respectively, for the true positives, false positives, and false negatives of class *i*. In our experiment, *n* represents the number of subjects. In the Equation (12), $p_{o}$ is the accuracy of our model, and $p_{e}$ denotes the hypothetical probability of chance agreement.

**Figure 7:**
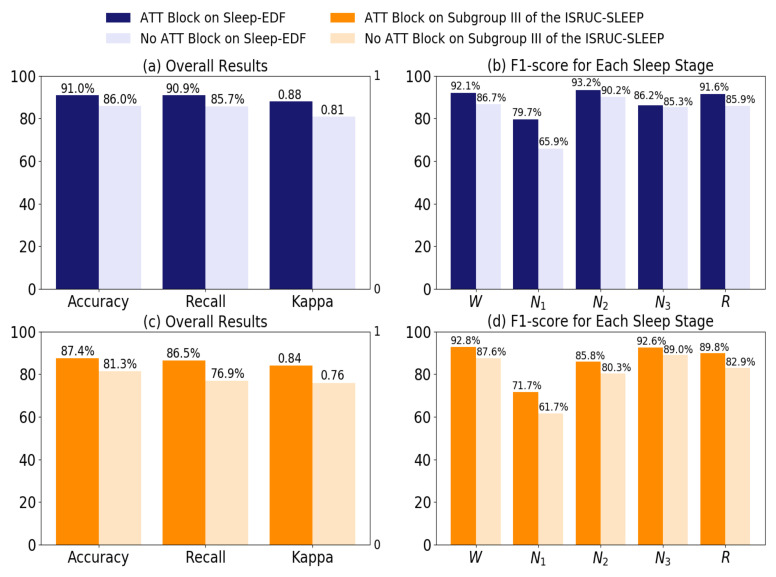
The comparison result of introducing ATT blocks and no ATT blocks. We employ the sleep-EDF dataset to obtain the comparison results, as shown in sub-figure (**a**) and sub-figure (**b**). The sub-figure (**c**) and sub-figure (**d**) present the performance comparison of introducing ATT blocks and no ATT blocks on the subgroup III of the ISRUC-SLEEP dataset. Obviously, the model with ATT blocks yields the best results in terms of all kinds of measuring metrics.

Macro-averaged performance obtained with the sleep-EDF dataset and the subgroup III of the ISRUC-SLEEP dataset are shown in Table 3 and Table 4. From Table 3, we can calculate that the macro-precision, macro-recall, and macro-F score are 87.4%, 90.9%, and 89.0%, respectively. From the Table 4, the macro-precision, macro-recall, and macro-F score are 86.6%, 86.5%, and 86.5%, respectively. In two different datasets, we obtain an accuracy of 91.0 % and 87.4 %, respectively. The Cohen’s kappa coefficients are 0.88 and 0.84, which is considered ideal as it outperforms the standard of 0.8 [52]. To validate the effect of introducing the ATT blocks, we use a 20-fold cross-validation on the sleep-EDF dataset and a 10-fold cross-validation on the subgroup III of the ISRUC-SLEEP dataset. The results of the comparisons are described in Figure 7. Figure 7 presents that the model with the ATT blocks performed better than the model without the ATT blocks in terms of overall accuracy and F1-score for each sleep stage. The performance has been significantly improved.

### 4.4. Comparisons with State-of-the-Art Models

To verify the superiority of our proposed model, we compare it with state-of-the-art models on the sleep-EDF dataset and the subgroup III of the ISRUC-SLEEP dataset. We use the same experimental settings to train all models. Compared to other baseline methods, our model outperforms significantly better than the state-of-the-art methods, as can be seen in Table 5 and Table 6. First, we consider previous works that utilize RNN and CNN to extract the spatial or temporal features for sleep stage classification. These non-GCN-based methods use grid data as input to high accuracy. However, EEGs, as non-Euclidean data, can be well processed by powerful GCNs. Therefore, we use two datasets to evaluate the performance of existing GCN-based methods and perform a comparative analysis.

As shown in Table 5 and Table 6, our proposed method presents the best overall performance compared to the state-of-the-art methods. The proposed method achieves the best accuracy (91.0% and 87.4%), the macro-F score (89.0% and 86.5%), and Kappa (0.88 and 0.84) on two datasets. For the subgroup III of ISRUC-SLEEP dataset, the proposed method provides the highest accuracy for each sleep stage. For the sleep-EDF dataset, our method achieves the highest accuracy for each sleep stage except for $N_{3}$ stage (sub-optimal). For $N_{1}$ stage, Table 5 and Table 6 show that the classification effect for $N_{1}$ stage on the two dataset is not as ideal as for the other sleep stages. It can be explained by two reasons. First, a number of samples in $N_{1}$ stage belong to the sleep transition period [54], thus the $N_{1}$ stage is misclassified into other stages. Second, the $N_{1}$ stage occupies a small proportion of the dataset. In particular, in the sleep-EDF dataset, the proportion of $N_{1}$ stage is only 6.7%.

**Table 6:** Comparison between our proposed method and the other state-of-the-art methods on subgroup III of ISRUC-SLEEP dataset across overall performance and F1-score for each sleep stage. The numbers in bold indicate the highest performance metrics of all methods and the underlined result is the sub-optimal result.

	Performance of Quality Assessment			Global F1-Score for Sleep Stages (%)				
Study (Year)	Accuracy (%)	Macro-F Score (%)	Kappa	*W*	$N_{1}$	$N_{2}$	$N_{3}$	*R*
Non-GCN-Based Methods								
Memar et al. [14] (2017)	72.9	70.8	0.65	85.8	47.3	70.4	80.9	69.9
Dong et al. [11] (2017)	77.9	75.8	0.71	86.0	46.9	76.0	87.5	82.8
DeepSleepNet [24] (2017)	78.8	77.9	0.73	88.7	60.2	74.6	85.8	80.2
RotSVM [13] (2018)	73.3	72.1	0.66	86.8	52.3	69.9	78.6	73.1
Phan et al. [21] (2018)	78.9	76.3	0.73	83.6	43.9	79.3	87.9	86.7
Chambon et al. [23] (2018)	78.1	76.8	0.72	87.0	55.0	76.0	85.1	80.9
Ghimatgar et al. [55] (2019)	75.7	73.5	0.69	85.0	49.4	75.4	83.1	74.8
Shen et al. [56] (2020)	81.7	80.2	0.76	89.1	62.5	80.4	86.5	82.4
GCN-Based Methods								
GraphSleepNet [29] (2021)	79.9	78.7	0.74	87.8	57.4	77.6	86.4	84.1
Jia et al. [30] (2021)	82.1	80.8	0.77	89.4	59.6	80.6	89.0	85.6
Our proposed method	**87.4**	**86.5**	**0.84**	**92.8**	**71.7**	**85.8**	**92.6**	**89.8**

## 5. Discussion

Sleep disorders are highly prevalent in the world. Especially in the United States, nearly 25% of adults suffer from sleep disorders [57]. Sleep disorders not only affect the quality of life, but also lead to health problems, such as heart disease and stroke. For people with sleep disorders to obtain adequate sleep, they may require the help of an appropriate method for sleep stage classification. In this work, we use a combination of dynamic and static ST-GCN with inter-temporal attention blocks to automatically classify sleep stages. We first consider that the distribution of brain electrodes is characteristic of non-Euclidean data. After the addition of ATT blocks, the sleep stage classification network achieves better performance. This confirms that spatial and temporal correlations play an important role in the sleep stage classification. The obtained results suggest that our method is promising in detecting new abnormalities in sleep and continuously improving our understanding of sleep mechanisms.

The NREM stages are divided into three sleep stages ($N_{1}$, $N_{2}$, and $N_{3}$) and are associated with the depth of sleep. Research shows that the stage $N_{3}$ may affect the ability to learn new information and memory retention [58]. In simple terms, $N_{3}$ is the deepest sleep stage, which has the strongest repair function. Tafaro et al. [59] report a positive relationship between sleep quality and survival in centenarians. From our experiment, the proposed method shows excellent performance in classifying the stage $N_{3}$ compared with stages $N_{1}$ and $N_{2}$. Therefore, accurate detection of the stage $N_{3}$ provides an aid to long-term care, health and welfare services for the elderly. One study [60] shows that patients with $REM_{OSA}$ in REM sleep had a significantly more collapsed airway and better ventilatory control stability compared with NREM sleep. Moreover, as it is suggested that the increased proportion of $N_{3}$ stage may reveal a lower severity of OSA [61], our method can be used as an ancillary treatment.

There are some challenges in more generic terms. First, since the stage $N_{1}$ is a transition period between wakefulness and sleep, it is difficult to detect this stage correctly. The system should be improved for the diagnosis of sleep fragmentation, such as obstructive sleep apnea. Second, the dataset is not perfect due to human errors. As far as we know, sleep scoring is defined by sleep experts. It is inevitable that similar sleep stages may be incorrectly marked. Therefore, the question for many sleep stage classification networks is how to use high-quality sleep stage datasets for the training process. In the future, we will develop a sleep stage system that provides more human-like performance to overcome the above challenges.

## 6. Conclusions

In this work, we propose a combination of dynamic and static ST-GCN with inter-temporal attention blocks for automatic sleep stage classification. Spatial graph convolutions and temporal convolutions are used to model the EEG data. We use a combination of dynamic and static ST-GCN to capture the global context-enriched topology and employ temporal convolution with dilation to enlarge the temporal receptive field. Furthermore, to the best of our knowledge, we introduce the attention blocks for the first time in the field of sleep stage classification to model the relationship between different EEG channels, which can capture long-range dependencies for sleep stage classification. The comparative results indicate that our method has powerful capability and expressiveness in sleep stage classification. Therefore, we believe that our method could be a complementary tool to help scientists to monitor the sleep status of patients to initiate appropriate treatments. In the future, since our method is used for sleep stage classification based on EEGs, we will apply it to a broader range of other physiological signal classification tasks. 

## Figures and Tables

**Table 1:** Details of the number of sleep stages in the subgroup III of the ISRUC-SLEEP dataset and sleep-EDF dataset.

Dataset	*W*	$N_{1}$	$N_{2}$	$N_{3}$	*R*	Total
**Sleep-EDF**	7927	2804	17,799	5703	7717	41,950
**ISRUC-SLEEP**	1817	1248	2678	2035	1111	8889

**Table 2:** The hyperparameters of our experiment.

Hyperparameters	Value
Optimizer	Adam
Batch size	64
Number of training epochs	120
Learning rate	Initial learning rate is 0.001 and is decayed by 10 at the 30th, 60th, and 90th epoch.
Dropout probability	0.2
Layer number of ST-GCN	9
Reduction ratio	4
Numbers of output channel for ST-GCN	66, 66, 66, 132, 132, 132, 264, 264, 264

**Table 3:** The confusion matrix of our proposed method on the sleep-EDF dataset.

	Predicted Stage						
		*W*	$N_{1}$	$N_{2}$	$N_{3}$	*R*	**Total**
**Actual stage**	*W*	**7371**	214	94	147	101	7927
	$N_{1}$	53	**2496**	201	44	10	2804
	$N_{2}$	480	552	**16,019**	187	561	17,799
	$N_{3}$	147	93	249	**5123**	91	5703
	*R*	21	103	15	410	**7168**	7717
	**Total**	8072	3458	16,578	5911	7931	**41,950**

**Table 4:** The confusion matrix of our proposed method on the subgroup III of the ISRUC-SLEEP dataset.

	Predicted Stage						
		*W*	$N_{1}$	$N_{2}$	$N_{3}$	*R*	**Total**
**Actual stage**	*W*	**1682**	83	37	7	8	1817
	$N_{1}$	94	**878**	183	6	87	1248
	$N_{2}$	19	179	**2297**	158	25	2678
	$N_{3}$	4	3	122	**1905**	1	2035
	*R*	8	59	37	3	**1004**	1111
	**Total**	1807	1202	2676	2079	1125	**8889**

**Table 5:** Comparison between our proposed method and the other state-of-the-art methods on the sleep-EDF dataset across overall performance and F1-score for each sleep stage. The numbers in bold indicate the highest performance metrics of all methods and the underlined result is the sub-optimal result.

	Performance of Quality Assessment			Global F1-Score for Sleep Stage (%)				
Method (Year)	Accuracy (%)	Macro-F Score (%)	Kappa	*W*	$N_{1}$	$N_{2}$	$N_{3}$	*R*
Non-GCN-Based Methods								
Tsinalis et al. [20] (2016)	74.8	69.8	–	65.4	43.7	80.6	84.9	74.5
Tsinalis et al. [9] (2016)	78.9	73.7	–	71.6	47.0	84.6	84.0	81.4
DeepSleepNet [24] (2017)	82.0	76.9	0.76	84.7	46.6	85.9	84.8	82.4
SeqSleepNet [16] (2017)	81.2	74.6	0.73	74.1	46.9	86.9	81.2	83.8
Phan et al. [21] (2018)	82.3	74.7	0.75	77.3	40.5	87.4	86.0	82.3
IITNet [19] (2019)	84.0	77.7	0.78	87.9	44.7	88.0	85.7	82.1
SleepEEGNet [22] (2019)	84.3	79.7	0.79	89.2	52.2	86.8	85.1	85.0
TinySleepNet [25] (2020)	85.4	80.5	0.80	90.1	51.4	88.5	**88.3**	84.3
GCN-Based Methods								
GraphSleepNet [29] (2021)	84.2	81.0	0.79	83.2	69.0	88.4	74.9	89.6
Jia et al. [30] (2021)	86.4	84.1	0.82	85.5	75.3	89.8	80.4	89.3
Our proposed method	**91.0**	**89.0**	**0.88**	**92.1**	**79.7**	**93.2**	88.2	**91.6**

## Data Availability

Two public datasets are used in this study: (1) Sleep-EDF dataset is available at https://physionet.org/content/sleep-edf/1.0.0/, accessed on 18 April 2022. (2) The subgroup III of the ISRUC-SLEEP dataset is available at https://sleeptight.isr.uc.pt/, accessed on 18 April 2022.

## Abbreviations

The following abbreviations are used in this manuscript:

PSG	Polysomnogram
EEG	Electroencephalogram
EOG	Electrooculogram
EMG	Electromyogram
ECG	Electrocardiogram
REM	Rapid Eye Movement
R&K	Rechtschaffen and Kales
NREM	Non-Rapid Eye Movement
AASM	American Academy of Sleep Medicine
SVM	Support Vector Machine
GCN	Graph Convolutional Network
RNN	Recurrent Neural Network
CNN	Convolutional Neural Network
LSTM	Long Short-Term Memory
GRU	Gated Recurrent Unit
ST-GCN	Spatiotemporal Graph Convolutional Network
MSTGCN	Multi-View Spatial-Temporal Graph Convolutional Network
DE	Differential Entropy
TCN	Temporal Convolutional Network
MLP	Multi-Layer Perceptron
FC layer	Fully Connected Layer
PFST	Portuguese Foundation for Science and Technology
REM	Rapid Eye Movement
TP	True Positive
FP	False Positive
FN	False Negative
OSA	Obstructive Sleep Apnea
